# Estimation and Discrimination of Stochastic Biochemical Circuits from Time-Lapse Microscopy Data

**DOI:** 10.1371/journal.pone.0047151

**Published:** 2012-11-06

**Authors:** David Thorsley, Eric Klavins

**Affiliations:** 1 Department of Defense Biotechnology High Performance Computing Software Applications Institute, Telemedicine and Advanced Technology Research Center, U.S. Army Medical Research and Materiel Command, Fort Detrick, Maryland, United States of America; 2 Department of Electrical Engineering, University of Washington, Seattle, Washington, United States of America; Virginia Tech, United States of America

## Abstract

The ability of systems and synthetic biologists to observe the dynamics of cellular behavior is hampered by the limitations of the sensors, such as fluorescent proteins, available for use in time-lapse microscopy. In this paper, we propose a generalized solution to the problem of estimating the state of a stochastic chemical reaction network from limited sensor information generated by microscopy. We mathematically derive an observer structure for cells growing under time-lapse microscopy and incorporates the effects of cell division in order to estimate the dynamically-changing state of each cell in the colony. Furthermore, the observer can be used to discrimate between models by treating model indices as states whose values do not change with time. We derive necessary and sufficient conditions that specify when stochastic chemical reaction network models, interpreted as continuous-time Markov chains, can be distinguished from each other under both continual and periodic observation. We validate the performance of the observer on the Thattai-van Oudenaarden model of transcription and translation. The observer structure is most effective when the system model is well-parameterized, suggesting potential applications in synthetic biology where standardized biological parts are available. However, further research is necessary to develop computationally tractable approximations to the exact generalized solution presented here.

## Introduction

Developing an understanding of biological phenomena through modeling requires the notion of a state that captures the essential components of the system and a model that describes its essential functions. When a collection of cells is considered in aggregate, measurement noise is usually primarily responsible for complicating the problem of identifying state and model parameters in genetic networks. At the single-cell level, the presence of cellular variability in experimental data [1] introduces systemic noise that further complicates this problem. However, noise can be used as a tool in the identification process. Munsky et al. [2] demonstrate the power of using both transient and steady-state noise statistics in parameter identification, as using both types of statistics yields more information about cellular parameters than steady-state noise alone. Likewise, Dunlop et al. [3] use the averages of correlations in expression level to identify regulatory elements in *Escherichia coli*.

The stochastic phenomenon of systemic noise in individual cells can be detected by observing the variation that occurs during the growth the of isogenic colonies observed using time-lapse microscopy [4]. The movies produced by these methods do not provide a full measurement of the system’s state but instead provide measurements of only a few species, such as fluorescing proteins, and these data are corrupted by measurement noise. For a stationary chemical process, “stochastic monitoring” [5] is a Bayesian approach to estimating the value of a state given all prior measurements and a master equation describing the state’s evolution; however this approach requires that the stochastic process be both stationary and observed at all time points. Boys et al. [6] use Bayesian inference for parameter estimation of a stochastic chemical process when the populations of a subset of the species are observed at intermittent time points, but do not consider the more general problem of estimating the dynamically changing state. Suter et al. [7] estimate transcriptional switching rates in mammalian genes using a similar Bayesian approach. However, to our knowledge, the problem of performing state estimation on a general stochastic chemical kinetic process with intermittent observations and branching (modeling cell division) has not been addressed in the literature.

In the standard mesoscopic formulation of stochastic chemical kinetics [8], the trajectories generated by a reaction network define it as a jump process, where the state of the system remains constant except at discrete points in time corresponding to the firing of reactions. As such, a stochastic chemical kinetic system under limited observation can be considered as a class of hidden Markov model or partially observed discrete-event system [9]. Several methods have been proposed for state estimation, identification, and diagnosis of partially observed stochastic discrete-event systems [10], [11] and for discrete-event systems with timing information [12]. By constructing an observer for stochastic chemical kinetics systems based on discrete-event systems, we can address the problems of state estimation and model identification in a general unified framework.

In this study, we propose an observer-based method for estimating system states, estimating parameters, and discriminating between mechanisms from a single colony of cells observed through time-lapse microscopy. We derive equations for calculating the posterior probability distributions for states and parameters from the observation of both a single cell and a complete colony. We derive necessary and sufficient conditions that specify when a set of models can be distinguished from each other using our method. We illustrate our approach by analyzing the Thattai-van Oudenaarden model [13], a standard model of transcription and translation.

## Results

### Stochastic Modeling

We consider a single-celled organism as a single, well-mixed compartment. Consider a reaction network in a chamber that satisfies the standard assumptions of stochastic chemical kinetics [8] and contains a set of 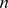 species 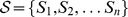 that interact along a set of 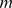 reaction channels 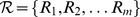. The state of the reaction network at time 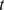 as a 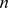-dimensional vector 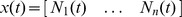, where 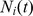 denotes the population of the species 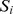 at time 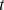. The state space 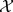 of the reaction network is countable and we index the states as 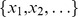. By enumerating the states we can construct a continuous-time Markov chain to describe the reaction network, consisting of the state space 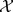, a transition rate matrix 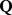, and an initial probability distribution 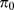. The transition rate matrix 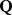 is constructed from the functions that define the rates of the reaction channels and these functions need not be linear. The 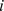
th element of the probability density vector 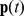 is the probability that the state of the network is 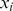 at time 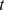. The probability density vector evolves according to the chemical master equation 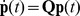, with initial condition 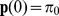. Many reaction network models permit the population of at least one species to grow without bound; for these networks, 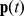 is an infinite-dimensional vector and 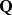 is an infinite-dimensional matrix. In this paper, we assume that for each species 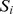, we can choose a value 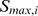 such that the probability of the population of 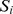 ever exceeding 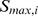 is negligibly small. We then disallow the firing of reaction channels that allow the population of each species to exceed the chosen maximum value. The size of the state space under these assumptions is 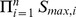, a very large but finite number.

The system is observed at a sequence of time points 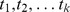; each time point corresponds to the capture of a time-lapse microscopy image. At each 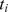 we observe the output 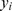; this quantity can be scalar- or vector-valued (corresponding to one-colour and multi-colour experiments, respectively). For each possible output value 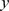 and each state 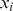, we define the probability density 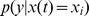. We construct the observation density vector 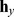 for each output by setting the 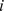
th element to 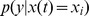. We also consider an idealized situation in which the system is observed continually on the interval 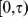 and all observations are noise-free, i.e. 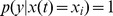 for some value of 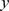, and 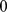 for all other values.

### State Estimation

The first problem we consider we call the forward problem. The objective of this problem is to the find the *a posteriori* probability distribution vector of the reaction network at a time 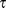, given the sequence of observations up to time 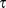. For each 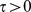, we set 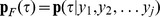, where 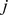 is the largest index such that 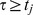. The dynamic evolution of 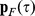 is described by the hybrid system.
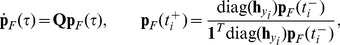
(1)where the left-hand equation describes the continuous evolution of 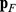 between observations and the right-hand equation describes the discrete change in 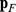 when an observation occurs. A full derivation of this system is given in Section 1 of Supporting Information S1.

For the idealized case of continual, noise-free observation, we can also describe the dynamic evolution of 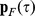 as a hybrid system. To do so, we must first define, for each pair of outputs 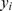 and 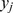, the matrix 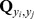. An element of 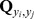 is equal to the corresponding element of 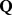 if the state associated with the row has output 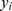, and the state associated with the column has output 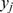. All the other elements of 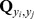 are equal to zero. The idealized forward observer is described by the hybrid system.
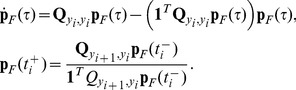
(2)


In the idealized case, the observed trajectory is a jump process with constant output between jumps. The left-hand equation describes the behavior of the system while the output is continually observed to be 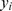. The second equation describes the change in the probability distribution when a change in output from 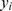 to 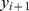 occurs. A full derivation of this system is also given in Section 1 of Supporting Information S1.

The structure of the “forward observers” uses the “predict-and-update” approach for observers found in control theory, such as the Kalman filter [14]. Between observation, the observer updates the probability distribution of the state using the chemical master equation. When an observation occurs at time 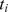, the probability mass function is re-weighted according to how likely each state was to have generated the observed output 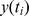.

The expected value taken with respect to the distribution 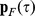 is the minimum mean-square error (MMSE) estimate of the state given the sequence of observations up to time 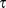. Whenever a new observation is taken, there is a discontinuous jump in the probability distribution and the MMSE estimate as the new information is incorporated. This jump occurs because the quantity 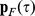 does not anticipate the arrival of new information; when a new observation is made, there is a discrete change in the information available to the forward observer and thus a discontinuity.

The forward observer thus computes the probability distribution of the current state of a process while an experiment is on-line. The second problem we consider is the related “backward” problem of finding the *a posteriori* probability distribution vector of the reaction network at a time 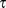 given the entire sequence of observations. For each 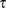, we define 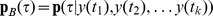 as the quantity we wish to calculate.

Given the results of the forward observer for discrete observations (Eq. 1), the probability 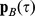 can be calculated for any 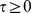 using the “backward observer”.
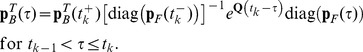
(3)


The names “forward observer” and “backward observer” are taken from the direction of calculation in time; the forward probability is calculated starting at 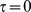 and ending at 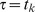; the backward probability equation is initialized with 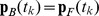 and then calculated backwards in time ending at 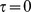.

**Figure 1:**
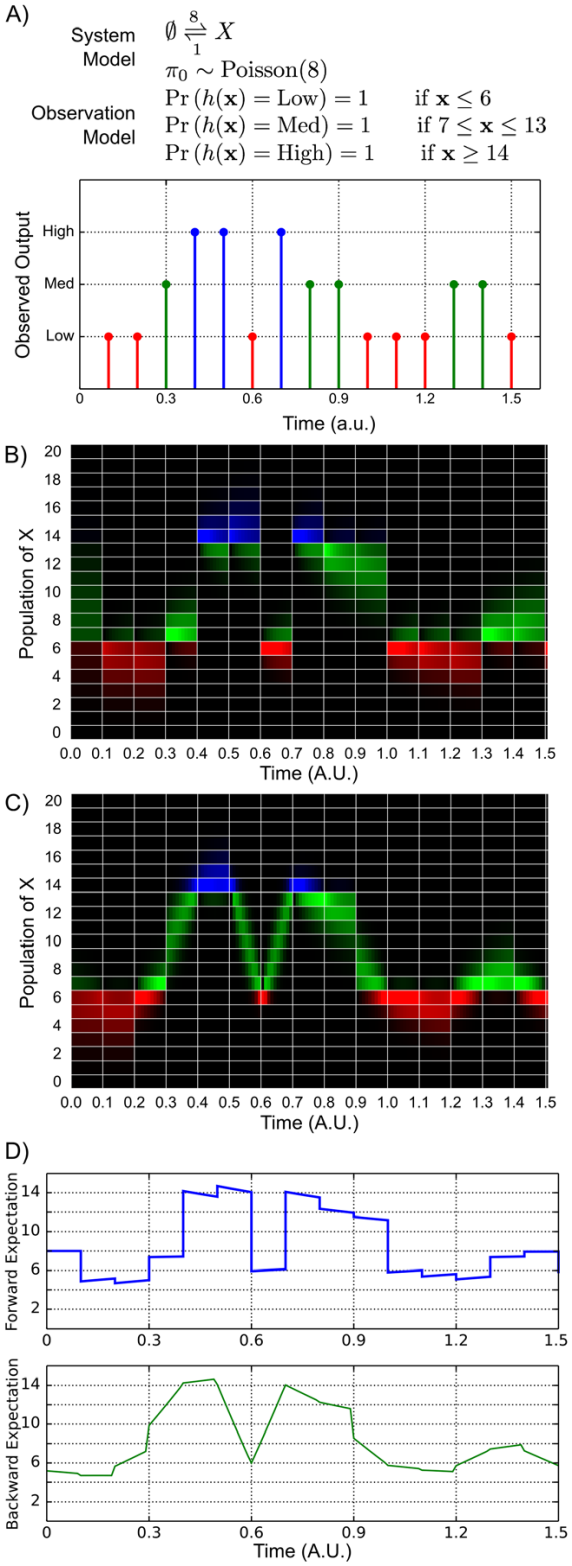
Implementation of the observer algorithms. (a) Inputs to the observer algorithm. (i) The system model consists is a birth-death reaction of a single species 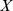. The initial distribution is the steady-state of this reaction network, a Poisson distribution with parameter 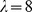. (ii) The sensor model partitions the state space into three sections with deterministic outputs. If the population of 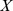 is less than or equal to 6, the observed output is “LOW”; if the population is between 7 and 13, the output is “MEDIUM.” Otherwise the output is “HIGH.” (iii) A sample time series trajectory of observed output. The process is observed intermittently with observations taken every.1 time units. (b) The time-varying probability distribution of the state estimate generated by the forward algorithm. At each observation point, there is a discontinuity in 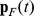 as new information is incorporated into the estimated probability mass function. The forward algorithm estimate lags the data, as it does not anticipate the values of future outputs. (c) The time-varying probability distribution of the state estimate generated by the backward algorithm. At each observation point, the state estimation is non-differentiable, but continuous. (d) Comparison of the evolution of the forward expectation 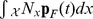 and the backward expectation 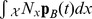. The shaded area indicates plus or minus one standard deviation.

Figure 1 shows the application of the forward and backward observer algorithms to a single-species birth-death process where the measured output of the system is a coarse-grained estimate of the population as “low,” “medium,” or “high.” Panel 1a shows a sample output from the system. Panels 1b and 1c show the outputs from the forward and backward observers, respectively, and indicate that discontinuities in 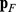 at each observation time are smoothed away in the backward probability distribution 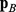. Panel 1d shows the expected *a posteriori* value of the species population from both observers.

### Model Discrimination

The forward and backward observer algorithms used to determine the state of the cellular process can, with a straightforward modification, also be used to distinguish between a finite set of candidate models of the process. These models can have different reaction structures or they can contain the same set of reaction channels but have differing reaction rates. Suppose that we wish to discrimate between a finite set of 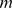 models 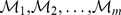. If the rate matrix associated with the model 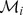 is 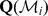, then running the observer algorithms using the block diagonal system matrix.
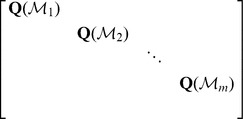
calculates the probability of each model given the sequence of observations. The model index is treated as a state of the system that cannot change with time; the probability distribution over the model space can then be calculated using the forward and backward observer algorithms. When the observer algorithms are applied to the block diagonal matrix, the set of states corresponding to a modele more likely to produce the observed trajectory increases in probability, while the set of states corresponding to a model less likely to produce the trajectory decreases in probability.

### Integrating Lineages

When a single cell grows into a colony of isogenic cells, different daughter cells will produce different sequences of observations due to the inherent stochasticity in both the chemical reaction and the observation process. If the observer algorithms are used for model discrimination on each daughter cell separately, they will produce differing probability distributions over the model space and likely disagreement as to the most likely model.

The final problem we consider is integrating the observations from many different cells that are all descendants of a single ancestral cell. Denote the single ancestral cell by 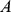 and the set of observations made on this cell before it divides by 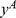. 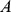 divides into two daughter cells that are themselves ancestors of two new lineages, which we denote by 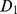 and 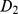; the observations made along these two sublineages are 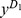 and 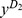, respectively. Our objective is to calculate the 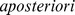 probability 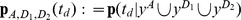, that is, the probability distribution vector for each cell given all the observations in the lineage.

Denote by 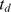 the time at which the ancestral cell divides. If we assume that each molecule in the reaction has equal probability of appearing in either of the two daughter cells after division, the probability distribution vector at the division time can be expressed as
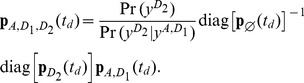
(4)


**Figure 2:**
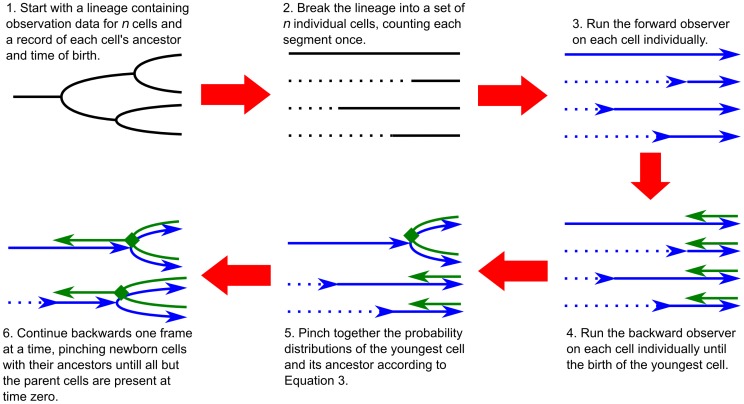
Procedure for integrating lineages. (a) Run the forward observer on each cell, breaking up the lineage so that each segment is only counted once. (b) Run the backward observer on each cell and “pinch” together a cell and its ancestor when the birth of a cell is observed. One forward and backward sweep of the lineage determines the posterior probability distribution 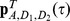 for 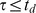, where 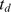 is the first division time. Calculating this probability distribution is sufficient for parameter estimation. To calculate posterior state estimates for 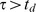, run an additional forward sweep on each cell, integrating the colony estimate with the results of the forward and backward observer.

The first factor, 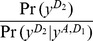 is a constant and the second factor 
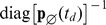
 is the *a priori* probability distribution vector at 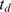, which can be calculated using the master equation. The last two factors are the probability density vectors calculated by dividing the total lineage into two sublineages 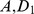 and 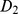. Each of these two sublineages has fewer cells than the original lineage and a first division time that occurs after 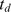. Because each of these sublineages is smaller than the original lineage, we can calculate 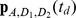 using a divide-and-conquer algorithm, as described in Figure 2.

Once the *a posteriori* probability distribution vector at 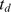 is calculated, we can calculate the probability distribution for all times less than 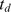 using the backwards observer.
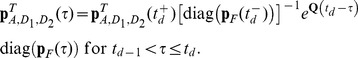
(5)


To find the probability distribution for the state of each cell at times after 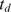, we use the results of running the forward and backward observers on each of the individual cells and run a forward sweep on each cell, starting with 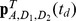. For each cell, the probability distribution is updated according to the equation



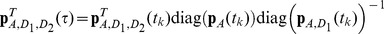


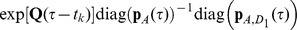
Details of the derivation are found in the Section 1 of Supporting Information S1.

### Identifiability of Models

When performing an experiment in order to conduct model discrimination, an important question to answer beforehand is to determine whether or not the models are identifiable, i.e., regardless of what outputs are observed, will it be possible to converge on a point estimate in the model space?

Consider a set of models 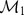, 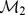, 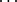, 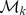. Suppose that the true underlying model is 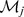. The set of models 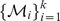 is distinguishable if for all 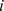, the following property holds: for all 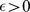 and 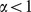, there exists a time 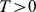 such that for all 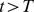,
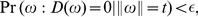
(6)where 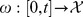 is a trajectory generated by the underlying model and 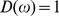 if there exists an 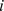 such that 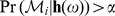 and 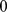 otherwise. If none of the models in the set describes the true system, then, if this condition holds, the model 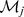 is distinguishable as the “best approximation” of the true system in that it is most likely to have produced the observed output.

For a stochastic chemical reaction network with reversible transitions in which the probability that any of the species populations increase without limit is zero, the underlying continuous-time Markov chain is positive recurrent and thus there exists a unique steady-state distribution 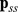 [15]. However, even though the unobserved chain has an *a priori* steady-state distribution, neither the forward observer 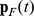 nor the backward observer 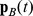 reaches a steady-state as 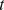 tends to infinity, because new observations result in discrete jumps in their evolution.

For simplicity of presentation, we consider the case where where each state 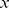 generates a single noiseless output 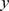. For each output, we can then describe 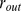, the rate at which a transition from a state with the output 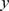 to a state with a different output occurs, as
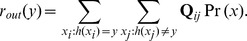



The output rate 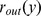 is dependent on the probability distribution 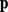 and thus varies as a function of 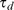, the dwell time of the system in the set of states with output 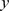. When the unobserved chain is in its *a priori* steady-state distribution, 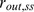, the value of 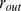 at steady state, does not depend on the entire output history, but instead depends only on the current output 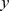, the previous output 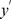, and the dwell time in the current output 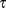. As a result, it follows that two models 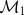 and 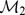 are indistinguishable if and only if
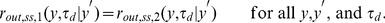
(7)


The proof of this theorem is included in Section 2 Supporting Information S1. The proof also demonstrates that the distinguishability of models does not, in theory, depend on the frequency of observation. However, because the distinguishability condition is an asymptotic condition that allows for an unlimited amount of time to distinguish between the models, more frequent observation is likely to lead to faster model discrimination in practical situations. Determining the rate at which discrimination occurs is intractable in general, however, Komorowski et al. [16] provide a solution for this problem in the case where the continuous-time jump Markov process is approximated using the linear noise approximation.

### Application to the Thattai-van Oudenaarden Model

The Thattai-van Oudernaarden model [13] is a simple model of stochastic transcription, translation and degradation. It consists of three species: 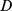 (DNA), 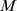 (messenger RNA), and 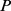 (protein). The four reactions in the model are



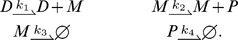



We denote this model by 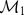 and set the rates as 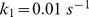, 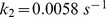, 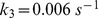, 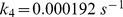 [17]. We select the initial conditions 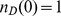, 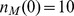, and 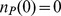.

**Figure 3:**
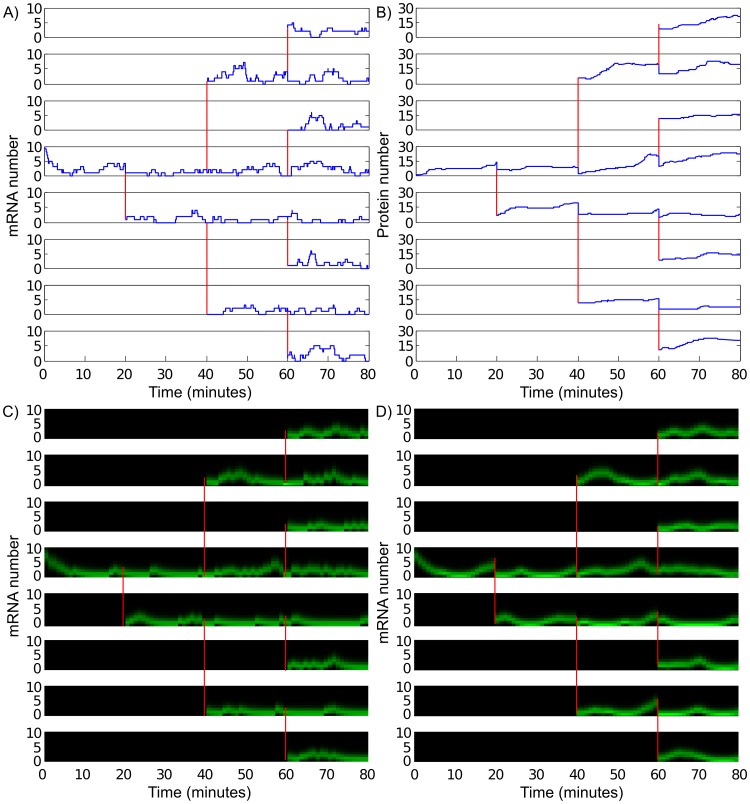
Using the observer to estimate mRNA population. (a) The unobserved mRNA population from a Gillespie SSA run of a colony where cells divide every 20 minutes and the system is observed every minute. (b) The observed protein number from the same Gillespie SSA run. (c) The estimate of the mRNA population in each cell as a function of time generated by the forward observer. (d) The estimate of the mRNA population in each cell as a function of time generated by the backward observer. In this example, we assumed that, when the cell divides, each molecule of mRNA and protein was equally likely to join both daughter cells. Each cell’s ancestor is the cell lineage is indicated by a red vertical line connecting the plot for a daughter cell to that of its mother cell. Brighter shades of green indicates mRNA populations that are more probable.

Panels (a) and (b) of Figure 3 show a sample trajectory for a colony from model 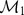, generated using the Gillespie stochastic simulation algorithm [18]. Panel (a) shows the dynamics of species M and panel (b) shows the dynamics of species P. The population of species D does not change as a result of any of the reactions and is not shown. We assumed the cells divide every 20 minutes. When a cell divides, we assumed that a copy of D is made so that there is one DNA strand in each cell in the colony at all times. We also assumed that each molecule of M and P is equally likely to join both daughter cells.

Figure 3(c) shows the estimate of the population of M generated by the forward observer for each cell in the colony with a sampling time of one minute. This estimate was generated by implementing Eq. 1 and the top three panels of Fig. 2. Each time a cell divides at a time 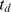, the probability mass function for each of the daughter cells at time 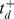 immediately following cell division was calculated from the probability mass function at time 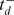 by the equation
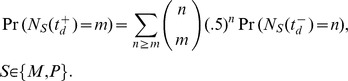



Figure 3(d) shows the estimate of the population of M generated by the backward observer for each cell in the colony. This estimate was generated by implementing Eqs. 3–5 and the bottom three panels of Figure 2. Each time a cell divides at time 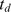, we “pinched” together the probability mass functions of the daughter cells by applying Eq. 3 to determine the probability mass function as 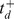. We then calculated the estimate of mRNA population at time 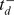 using the equation
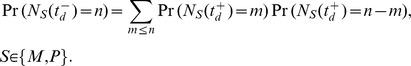



Note that the output of the backwards observer is continuous with time except at each multiple of 20 minutes when cell division occurs.

**Figure 4:**
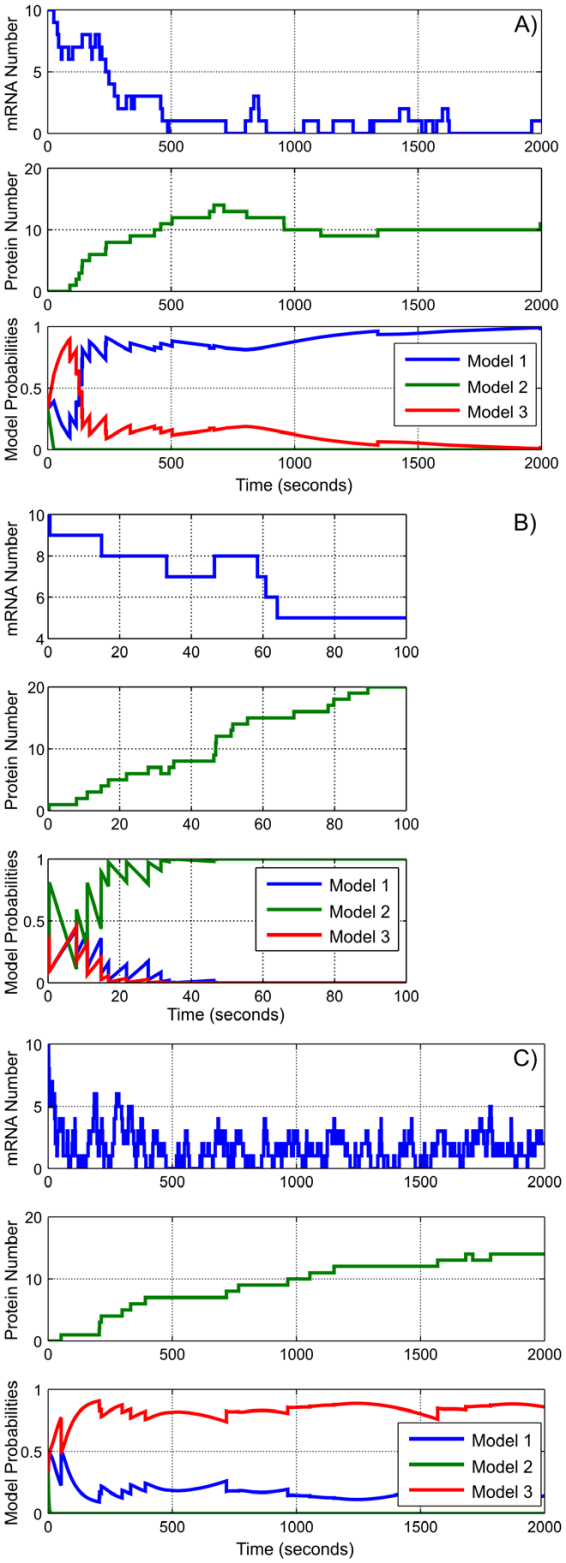
Using the observer to discriminate between models. We consider three models in this figure: 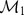, a standard Thattai-van Oudenaarden model of transcription and translation, 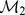, a structurally-identical model in which the rates of protein production and degradation are increased by a factor of 10, and 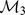, another structurally-identical model in which the rates of messenger RNA production and degradation are increased by a factor of 10. Each subfigure contains three plots: the unobserved mRNA number from a stochastic simulation run (top), the observed protein number from the same SSA run (middle), and the idealized observer estimates as to the posterior probabilities of each model (bottom). The system is observed continually. (a) Results from simulated data generated from 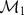. (b) Results from simulated data generated from 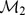. Note that the observer decision is very quick in this scenario, as the observable protein dynamics in 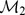 differ from those of both 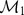 and 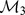. (c) Results from simulated data generated from 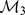. Note that despite the similar appearance of the protein number trajectory here and in (a), the observer is able to determine which unobservable mRNA dynamics are responsible for the observed behavior.

We also demonstrate how to use the idealized forward observers for model discrimation. Panel (a) of Figure 4 shows a sample trajectory for a single cell generated using the SSA from the same model 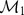.

We consider two alternate models. The first, 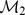, has the same structure as 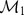, but the rates of protein production and degradation are increased by an order of magnitude. The four reactions in 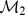 are



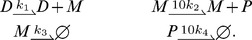



A Gillespie simulation from this model is shown in Panel 4(b). The second alternative model, 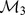, also has the same structure as 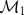, except here the rates of mRNA production and degradation are increased by an order of magnitude. The reactions in 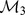 are


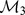
, also has the same structure as 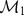, except here the rates of mRNA production and degradation are increased by an order of magnitude. The reactions in 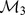 are
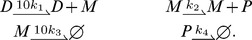



A Gillespie simulation from this model is shown in Panel 4(c).

These three models all have the same steady-state distribution, so, in order to distinguish them, it is necessary to use transient data. As an example, we assume that the generated trajectories are observed continually and construct the block-diagonal observer matrix for model discrimination. According to the distinguishability condition, all three models are distinguishable from each other. Because the system is observed continually, we use the observer equations from Eq. 2.

Consider the trajectory generated by 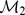. As Panel 4(b) shows, the observed protein number fluctuates rapidly as the translation rates are faster in 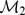 than they are in the other model. Panel 4(b) (bottom plot) shows that the observer can distinguish 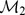 from the other candidate models with near certainty within 100 seconds.

The trajectories generated by 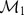 and 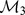 produce similar protein number trajectories, but the unobservable mRNA dynamics vary by an order of magnitude. In the limit as 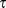 grows large, the quantities 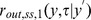 and 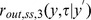 approach the same value, because the mRNA number approaches the same steady-state distribution for both models. However, when 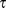 is small, the distributions of the mRNA number are far from steady-state and thus 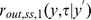 and 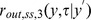 vary. If the observable protein number changes when 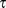 is small, this change provides information that can be used to determine the speed of the hidden mRNA dynamics. As Panels 4(a) and 4(c) (bottom plots) show, the observer is able to distinguish between the two models within 2000 seconds.

## Discussion

A fundamental issue limiting our understanding of the dynamics of cellular networks is that of sensing. Fluorescent proteins, the most commonly used sensors in the laboratory today, have multiple limitations that make their indiscriminate use unadvisable [19]. These limitations include the limited palette of visible fluorescence due to overlapping emission and excitation spectra, which means that we can only observe, at most, three of four tagged proteins in any one experiment [20]. Also, the effect of photobleaching in time-lapse fluorescence measurements limits the number of times that dynamic data can be collected for any one cell. Finally, the production of fluorescent proteins places a metabolic load on the cell that does not lead to an increase in fitness, so cells that fluoresce and provide the experimenter with information can be outcompeted by those that do not.

In light of these issues with the current state-of-the-art in sensing, it is imperative that we develop methods to extract as much information as possible out of the limited measurement techniques we do have available to us. Model-based approaches allow the experimenter to extract additional information and meaning from limited data indirectly through the design of observation algorithms and platforms, but require a reasonable amount of confidence in the accuracy of both the model of the system being studied and the experimental environment in which the measurements are being carried out.

In this paper, we develop a general theoretical method for observing the state of a process inside a single-celled organism based on the assumptions of stochastic chemical kinetics. This algorithm takes as its input a sequence of observations and outputs a probability distribution over the state space or parameter space of the system. We present forward observer algorithms for both discrete and continual observations, which estimate the state of the system using only past data, a backward observer algorithm, that estimates the state using all the collected data, including future data, and a colony algorithm for integrating the different trajectories generated by daughters of the same ancestral cell. For simplicity, in this paper, we presented the algorithm using the notation of finite-state, time-invariant Markov chains. However, the observer approach described here is more generally applicable as long as the system model chosen provides a method of constructing the transition semigroup [5], [15] that corresponds to the assumptions made by the modeler. Provided that the transition semigroup can be calculated or estimated efficiently, the fundamental concepts of the observer approach developed here can be extended to time-varying systems, systems with infinite state spaces, and larger systems that can be solved using approximate chemical master equation [21] or simulation methods [22].

The two main limitations of our method are the “curse of dimensionality” and the need for accurate parameterization of the system and sensor models. The state of a stochastic chemical kinetic system is a 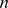-dimensional vector, where 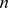 is the number of species in the equation. As a result, the size of the state space of the underlying continuous-time Markov chain is exponential in 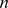 and the chemical master equation cannot be directly solved if 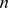 is large. For larger systems, it will be necessary to develop methods of approximating the chemical master equation solution that will likely be specific to the class of reaction network under consideration.

The accuracy of the posterior probability distributions calculated by the observer algorithms is dependent on the accuracy of the parameters in both the system model and the sensor model. Therefore, the applicability of our method is limited by the experimentalist’s ability to determine not only reaction rates and network structures in the system being studied, but also the dynamical properties of the type of sensor (e.g., fluorescent proteins) being used. Due to these limitations, we expect that our approach will be of more interest to synthetic biologists, who typically study systems with fewer parameters than those studied by systems biologists. However, the need for accurate parameter values is a problem that needs to be addressed in this approach for systems of all sizes.

We demonstrated the algorithm on the Thattai-van Oudenaarden model of transcription and translation. Because this model contains only two species whose populations change with time, it is possible to solve the chemical master equation with negligible truncation error and thus to make computationally tractable estimates of the unobservable mRNA population without resorting to more advanced approximation techniques. Furthermore, there exists a standard set of parameters for this model, allowing us to sidestep the problem of inaccurate parameterization. By applying the necessary and sufficient conditions for models to be distinguishable from each other, we can determine in advance that the observer is potentially effective in detecting differences in both the protein and the mRNA dynamics, although more time is needed to distinguish models with different hidden mRNA dynamics from those with different visible protein dynamics. However, because the distinguishability result describes the asymptotic behavior of the observer, it does not guarantee that the systems can be distinguished in a reasonable amount of time. Further research is required to quantify the rate of distinguishability for general stochastic chemical reaction networks.

Hopefully, as the state-of-the-art in computation power and experimental power continues to grow, the method described in this paper can be built upon to uncover knowledge of the dynamics of finer details of cellular operation. To address the realistic situation where it is not possible to accurately parameterize the model before applying the observer, future theoretical development of the observer algorithm will include the development of adaptive observer algorithms to simultaneously estimate the parameters and the states. To apply the observer to the estimation of unknown quantities when a parameterized model is available, we envision the following general procedure. First, select a few cells or colonies on which the observer algorithm has been applied for state estimation, and then perform a more expensive experimental test in order to verify the observer’s predictions. Once satisfied with the observer’s performance, the experimenter can then use the observer for high-throughput analysis on live cells, taking advantage of its indirect sensing method to perform experiments more rapidly and cost-effectively.

## Materials and Methods

All simulations were carried out in MATLAB R2011B. Code is available in the supporting information.

## Supporting Information

Supporting Information S1 **Contains proofs of the results in the main text.**
(ZIP)

Supporting Information S2 **Contains codes to reproduce the figures in the paper.**
(PDF)

## References

[1] Shahrezaei V, Swain P (2008) The stochastic nature of biochemical networks. Current Opinion in Biotechnology 19: 369–374. 18662776 10.1016/j.copbio.2008.06.011

[2] Munsky B, Trinh B, Khammash M (2009) Listening to the noise: random fluctuations reveal gene network parameters. Molecular Systems Biology 5. 10.1038/msb.2009.75 PMC2779089 19888213

[3] Dunlop M, Cox III S, Levine J, Murray R, Elowitz M (2008) Regulatory activity revealed by dynamic correlations in gene expression noise. Nature Genetics 40: 1493–1498. 19029898 10.1038/ng.281 PMC2829635

[4] Locke J, Elowitz M (2009) Using movies to analyse gene circuit dynamics in single cells. Nature Reviews Microbiology 7: 383–92. 19369953 10.1038/nrmicro2056 PMC2853934

[5] Van Kampen NG (2007) Stochastic Processes in Physics and Chemistry, Third Edition (North-Holland Personal Library). North Holland.

[6] Boys R, Wilkinson D, Kirkwood T (2008) Bayesian inference for a discretely observed stochastic kinetic model. Statistics and Computing 18: 125–135.

[7] Suter DM, Molina N, Gatfield D, Schneider K, Schibler U, et al (2011) Mammalian genes are transcribed with widely different bursting kinetics. Science 332: 472–474. 21415320 10.1126/science.1198817

[8] McQuarrie D (1967) Stochastic approach to chemical kinetics. Journal of Applied Probability 4: 413–478.

[9] Cassandras C, Lafortune S (1999) Introduction to Discrete Event Systems. Boston, MA: Kluwer Academic Publishers.

[10] Thorsley D, Teneketzis D (2005) Diagnosability of stochastic discrete-event systems. IEEE Trans-actions on Automatic Control 50: 476–492.

[11] Athanasopoulou E, Li L, Hadjicostis C (2010) Maximum likelihood failure diagnosis in finite state machines under unreliable observations. IEEE TAC 55: 579–593.

[12] Hashtrudi Zad S, Kwong R, Wonham W (2005) Fault diagnosis in discrete-event systems: incor-porating timing information. IEEE Transactions on Automatic Control 50: 1010–1015.

[13] Thattai M, Van Oudenaarden A (2001) Intrinsic noise in gene regulatory networks. Proceedings of the National Academy of Sciences of the USA 98: 8614–8619. 11438714 10.1073/pnas.151588598 PMC37484

[14] Kalman RE (1960) Contributions to the theory of optimal control. Boletin de la Sociedad Matem-atica Mexicana 5: 102–119.

[15] Brémaud P (1999) Markov Chains: Gibbs Fields, Monte Carlo Simulation and Queues. New York: Springer.

[16] Komorowski M, Costa MJ, Rand DA, Stumpf MPH (2011) Sensitivity, robustness, and identifi-ability in stochastic chemical kinetics models. Proceedings of the National Academy of Sciences 108: 8645–8650. 10.1073/pnas.1015814108 PMC3102369 21551095

[17] Hayot F (2011) Simulations of stochastic biological phenomena. Science Signaling 4: tr13. 21954294 10.1126/scisignal.2001973 PMC3225182

[18] Gillespie D (1977) Exact stochastic simulation of coupled chemical reactions. Journal of Physical Chemistry 81: 2340–2361.

[19] Ball DA, Marchand J, Poulet M, Baumann WT, Chen KC, et al (2011) Oscillatory dynamics of cell cycle proteins in single yeast cells analyzed by imaging cytometry. PLOS ONE 6: e26272. 22046265 10.1371/journal.pone.0026272 PMC3202528

[20] Davidson M, Campbell R (2009) Engineered fluorescent proteins: innovations and applications. Nature Methods 6: 713–717. 19953681 10.1038/nmeth1009-713

[21] Munsky B, Khammash M (2006) The finite state projection algorithm for the solution of the chemical master equation. Journal of Chemical Physics 124: 044104. 16460146 10.1063/1.2145882

[22] Gillespie DT (2007) Stochastic simulation of chemical kinetics. Annual Review of Physical Chemistry 58: 35–55. 10.1146/annurev.physchem.58.032806.104637 17037977

