# Supplementary material for: Estimation and Discrimination of Stochastic Biochemical Circuits from Time-Lapse Microscopy Data
Source: PLoS One. 2012 Nov 6;7(11):e47151. doi: 10.1371/journal.pone.0047151 (PMC3491022; doi:10.1371/journal.pone.0047151)
Supplement: Supporting Information S1 — Contains proofs of the results in the main text. (ZIP) [file pone.0047151.s001.zip]

Supplementary Material for "Estimation and Discrimination of Stochastic Biochemical Circuits From Time-Lapse Microscopy Data"

David Thorsley[1,*], Eric Klavins[2]

1 Department of Defense Biotechnology High Performance Computing Software Applications Institute, Telemedicine and Advanced Technology Research Center, U.S. Army Medical Research and Materiel Command, Fort Detrick, MD, USA

2 Department of Electrical Engineering, University of Washington, Seattle, WA, USA

* **E-mail: thorsley@u.washington.edu**

1 Derivation of Observer Equations

1.1 Derivation of Forward Observer

The derivation of the evolution of the probability distribution vector in the intermittent observation case is a continuous-time analogue to the approach used in [1] for stochastic discrete-event systems. For simplicity, we use the notion of finite-state Markov chains; an arbitrary biochemical network can be rationally approximated with a finite state space through the projection approach in [2]. We find $\mathbf{p}_F(\tau) = \mathbf{p}(\tau \mid y^k), t_k \leq \tau < t_{k+1}$ by decomposing the evolution of the *a posteriori* probability distribution into two domains. Between observation times, we show that $\mathbf{p}(\tau \mid y^k)$ evolves continuously according to the equation

$$\mathbf{p}(\tau \mid y^k) = e^{\mathbf{Q}(\tau - t_k)}\mathbf{p}(t_k \mid y^k). \tag{1}$$

At the times $t_1, t_2 \ldots t_n$ when an observation occurs, we show that $\mathbf{p}(t \mid y^k)$ evolves according to a discrete jump

$$\mathbf{p}(t_k^+ \mid y^k) = \frac{1}{\alpha_k}\mathbf{H}_{y_k}\mathbf{p}(\tau_k^- \mid y^{k-1}). \tag{2}$$

To describe the evolution of the state *between observations*, we condition of the value of the state at t_k, the time of the most recent observation, yielding the equation

$$\begin{aligned}
\Pr\left(x(\tau) = x_j \mid y^k\right) &= \sum_{x_i \in \mathcal{X}} \Pr\left(x(\tau) = x_j, x(t_k) = x_i \mid y^k\right) \\
&= \sum_{x_i \in \mathcal{X}} \Pr\left(x(\tau) = x_j \mid x(t_k) = x_i, y^k\right) \Pr\left(x(t_k) = x_i \mid y^k\right).
\end{aligned}$$

The right hand side of the above equation is the expression for calculating one element in a matrix multiplication. Constructing the vector $\mathbf{p}(\tau \mid y^k)$ from this expression yields

$$\mathbf{p}(\tau \mid y^n) = \begin{bmatrix} \Pr\left(x(\tau) = x_1 \mid x(t_k) = x_1\right) & \dots & \Pr\left(x(\tau) = x_1 \mid x(t_k) = x_m\right) \\ \vdots & \ddots & \vdots \\ \Pr\left(x(\tau) = x_m \mid x(t_k) = x_1\right) & \dots & \Pr\left(x(\tau) = x_m \mid x(t_k) = x_m\right) \end{bmatrix} \begin{bmatrix} \Pr\left(x(t_k) = x_1 \mid y^k\right) \\ \vdots \\ \Pr\left(x(t_k) = x_m \mid y^k\right) \end{bmatrix}, \tag{3}$$

$$= e^{\mathbf{Q}(\tau - t_k)} \mathbf{p}(t_k \mid y^k),$$

where the last line follows from the solution to the CME.

To calculate how the probability distribution updates discretely *at observation times*, we condition of the state of the process at a time $t_k - dt$ for small dt.

$$\begin{aligned}
\Pr\left(x(t_k^+) = x_j \mid y^k\right) &= \sum_{x_i \in \mathcal{X}} \Pr\left(x(t_k^+) = x_j, x(t_k - dt) = x_i \mid y^k\right) \\
\Pr\left(x(t_k^+) = x_j \mid y^k\right) &= \frac{1}{\Pr(y_k \mid y^{k-1})} \sum_{x_i \in \mathcal{X}} \Pr\left(x(t_k^+) = x_j, x(t_k - dt) = x_i, y_k \mid y^{k-1}\right) \\
\Pr\left(x(t_k^+) = x_j \mid y^k\right) &= \frac{1}{\alpha_k} \sum_{x_i \in \mathcal{X}} \Pr\left(x(t_k^+) = x_j, y_k \mid x(t_k - dt) = x_i\right) \Pr\left(x(t_k - dt) = x_i \mid y^{k-1}\right),
\end{aligned} \tag{4}$$

where $\alpha_k = \Pr(y_k \mid y^{k-1})$. For small dt, the quantity $\Pr(x(t_k^+) = x_j, y_k \mid x(t_k - dt) = x_i)$ reduces to $\mathbf{Q}_{ij} dt \Pr(h(x_j) = y_k)$ if $i \neq j$; if $i = j$, the term reduces to $(1 + \mathbf{Q}_{ii} dt) \Pr(h(x_i) = y_k)$. As $dt \to 0$, the first of these expressions goes to zero while the second goes to $\Pr(h(x_i) = y_k)$, which is equal to $(\mathbf{h}_{y_k})_i$.

Therefore

$$\Pr(x(t_k^+) = x_j \mid y^k) = \begin{cases} \frac{1}{\alpha_k}(\mathbf{h}_{y_k})_i \Pr(x(t_k^-) = x_i \mid y^{k-1}) & \text{if } i = j \\ 0 \quad \text{otherwise,} \end{cases}$$

For brevity, we define the matrix $\mathbf{H}_y = \text{diag}(\mathbf{h}_y)$. Writing the above expression in vector form yields

$$\mathbf{p}_F(t_k^+) = \frac{1}{\alpha_k}\mathbf{H}_{y_k}\mathbf{p}(t_k^- \mid y^{k-1}).$$

Because $\mathbf{p}_F(t_k^+)$ is a probability distribution, it follows that α_k is a normalization constant and thus, $\alpha_k = \mathbf{1}^T\mathbf{H}_{y_k}\mathbf{p}(t_k^- \mid y^{k-1})$.

Combining the results of the discrete and continuous cases yields

$$\mathbf{p}_F(\tau) = \frac{1}{\alpha_k}e^{\mathbf{Q}(\tau - t_k)}\mathbf{H}_{y_k}\mathbf{p}(t_k^- \mid y^{k-1}).$$

Similarly we can show that $\mathbf{p}_F(t_k^-) = \frac{1}{\alpha_{k-1}}e^{\mathbf{Q}(t_n - t_{n-1})}\mathbf{H}_{y_{n-1}}\mathbf{p}_F(t_{n-1}^-)$ for all $2 \leq n \leq k-1$. For $k = 1$, $\mathbf{p}(t_1^- \mid y^0) = e^{\mathbf{Q}\tau_1}\pi_0$ as a result of the *a priori* CME; because there are no observations before t_1, the conditional and unconditional distributions are equal. Combining all these results yields the desired result

$$\mathbf{p}_F(\tau) = \frac{1}{\alpha^k}e^{\mathbf{Q}(\tau - t_k)}\mathbf{H}_{y_n}e^{\mathbf{Q}(t_n - t_{n-1})}\mathbf{H}_{y_{n-1}}\ldots\mathbf{H}_{y_2}e^{\mathbf{Q}(t_2 - t_1)}\mathbf{H}_{y_1}e^{\mathbf{Q}(t_1)}\pi_0,$$

where $\alpha^k = \alpha_1\alpha_2\ldots\alpha_k$.

1.1.1 Hybrid System Representation

The derivation of the hybrid system representation is simpler when the observations are intermittent. Consider a small interval $[\tau, \tau + d\tau]$ on which no observations are made. Following the same procedure as in the case of continuous observations we obtain

$$\frac{d\mathbf{p}_F(\tau)}{d\tau} = \lim_{d\tau \to 0}\frac{1}{d\tau}e^{\mathbf{Q}d\tau}\mathbf{p}_F(\tau) - \mathbf{p}_F(\tau).$$

Using a linear approximation for $e^{\mathbf{Q}d\tau}$ for small $d\tau$ yields

$$\frac{d\mathbf{p}_F(\tau)}{d\tau} = \lim_{d\tau \to 0}\frac{1}{d\tau}(I + \mathbf{Q}d\tau)\mathbf{p}_F(\tau) - \mathbf{p}_F(\tau) = \mathbf{Q}\mathbf{p}_F(\tau).$$

Now consider the instant t_k whe an observation y_k is made. As noted above, for Equation 2 to conserve probability mass, the constant α_k must be equal to $\mathbf{1}^T\mathbf{H}_{y_k}\mathbf{p}_F(t_k^-)$. Therefore Equation 2 is equal to

$$\mathbf{p}_F(t_k^+) = \frac{\mathbf{H}_{y_k}\mathbf{p}_F(t_k^-)}{\mathbf{1}^T\mathbf{H}_{y_k}\mathbf{p}_F(t_k^-)}.$$

Writing the expression in a simplified hybrid systems notation and suppressing the argument yields the forward algorithm defined in the main paper.

1.2 Derivation of Idealized Forward Observer

In the idealized forward observer, observations are made continually and they are noise-free, i.e., each state x of the reaction network generates a unique output y. The observed trajectory is therefore constant for almost all time, and jumps between discrete output values when a reaction fires that changes the state to one that generates a different output. In the continual observation case, the quantity we wish to calculate is $\mathbf{p}_F(\tau) = \mathbf{p}(\tau \mid y^{[0,\tau)})$, where $y^{[0,\tau)}$ denotes the time course of observations from time zero to time τ.

We solve this problem by proceeding as in the discrete case with observations occurring every dt time units, and then taking the limit as $dt \to 0$. We follow the derivation for the behavior of the observer at discrete observation times, yielding the continuous analogue to Eq. 4:

$$\Pr\left(x(t+dt) = x_j \mid y^{[0,t+dt)}\right) = \frac{1}{\alpha_k}\sum_{x_i\in\mathcal{X}} \Pr\left(x(t+dt) = x_j, y^{[t,t+dt)} \mid x(t) = x_i\right) \Pr\left(x(t) = x_i \mid y^{[0,t)}\right), \tag{5}$$

where $\alpha_k = \Pr(y^{[t,t+dt)} \mid y^{[0,t)})$.

If dt is small enough that the probability of two or more jumps in the observation process in the interval $[t, t+dt)$ in negligible, then we need to consider only two cases. In the first case we set $h(x_j) = h(x_i) = y_i$. Then

$$\Pr\left(x(t+dt) = x_j \mid y^{[0,t+dt)}\right) = \begin{cases} (1+\mathbf{Q}_{ii}dt)\Pr\left(x(t) = x_i \mid y^{[0,t)}\right) & \text{if } i = j, \\ (\mathbf{Q}_{ij}dt)\Pr\left(x(t) = x_i \mid y^{[0,t)}\right) & \text{if } i \neq j \text{ and } h(x_j) = y_i, \\ 0 & \text{if } h(x_j) \neq y_i. \end{cases} \tag{6}$$

To express this equation in vector form, we define the matrix $\mathbf{Q}_{y_i,y_i}$ as equal to $\mathbf{Q}$ for the rows and columns corresponding to states with output y_i, and zero elsewhere. Using this notation, the three cases above can be combined into one vector equation

$$\mathbf{p}_F(t+dt) = \frac{1}{\alpha_k}\left(\mathbf{I} + \mathbf{Q}_{y_i,y_i}dt\right)\mathbf{p}_F(t).$$

For $\mathbf{p}_F(t+dt)$ to be a valid probability distribution, it follows that $\alpha_k = \mathbf{1}^T\left(\mathbf{I} + \mathbf{Q}_{y_i,y_i}dt\right)\mathbf{p}_F(t)$. Rearranging the terms in the above equation yields

$$\mathbf{p}_F(t+dt) - \mathbf{p}_F(t) = \mathbf{Q}_{y_i,y_i}dt\mathbf{p}_F(t) - \left(\mathbf{1}^T\mathbf{Q}_{y_i,y_i}dt\right)\mathbf{p}_F(t).$$

Dividing both sides by dt and taking the limit as $dt \to 0$ yields the idealized observer equation along a constant output,

$$\dot{\mathbf{p}}_F(t) = \mathbf{Q}_{y_i,y_i}\mathbf{p}_F(t) - \left(\mathbf{1}^T\mathbf{Q}_{y_i,y_i}\right)\mathbf{p}_F(t). \tag{7}$$

In the second case, we set $h(x_j) = y_j \neq y_i$. In this case,

$$\Pr\left(x(t+dt) = x_j \mid y^{[0,t+dt)}\right) = \begin{cases} \left(\mathbf{Q}_{ij}dt\right)\Pr\left(x(t) = x_i \mid y^{[0,t)}\right) & \text{if } h(x_j) = y_j, \\ 0 & \text{if } h(x_j) \neq y_j. \end{cases} \tag{8}$$

To express this equation is vector form, we define the matrix $\mathbf{Q}_{y_i,y_j}$ as equal to $\mathbf{Q}$ when the row corresponds to a state with output y_i and the column to a state with output y_j, and zero elsewhere. We then can write the vector equation

$$\mathbf{p}_F(t+dt) = \frac{1}{\alpha_k}\left(\mathbf{Q}_{y_i,y_j}dt\right)\mathbf{p}_F(t^-).$$

For $\mathbf{p}_F(t+dt)$ to be a valid probability distribution, it follows that $\alpha_k = \mathbf{1}^T\left(\mathbf{Q}_{y_i,y_j}dt\right)\mathbf{p}_F(t^-)$. Taking the limit as $dt \to 0$ from the right yields

$$\mathbf{p}_F(t^+) = \frac{\left(\mathbf{Q}_{y_i,y_j}dt\right)\mathbf{p}_F(t^-)}{\mathbf{1}^T\left(\mathbf{Q}_{y_i,y_j}dt\right)\mathbf{p}_F(t^-)}. \tag{9}$$

This equation describes the change in the probability distribution when a change in output is observed. Eqs. 7 and 9 together describe the structure of the idealized forward observer and appear as Eq. 2 in the

main text.

1.3 Derivation of Backward Algorithm

The backward algorithm smooths the state estimates given by the forward algorithm, producing a state estimate that is continuous in time. To find the posterior probability $\mathbf{p}_B(\tau) = \mathbf{p}(\tau \mid y^n)$ for $t_{k-1} \leq \tau < t_k$, we condition on the value of the state at t_k:

$$
\begin{aligned}
\Pr(x(\tau) = x_i, \mid y^n) &= \sum_{x_j \in \mathcal{X}} \Pr(x(\tau) = x_i, x(t_k) = x_j \mid y^n) \Pr(x(t_k) = x_j \mid y^n) \\
&= \sum_{x_j \in \mathcal{X}} \Pr(x(\tau) = x_i, x(t_k) = x_j \mid y^{k-1}) \Pr(x(t_k) = x_j \mid y^n) \\
&= \sum_{x_j \in \mathcal{X}} \frac{\Pr(x(t_k) = x_j \mid y^n)}{\Pr(x(t_k) = x_j \mid y^{k-1})} \Pr(x(t_k) = x_j \mid x(\tau) = x_i, y^{k-1}) \Pr(x(\tau) = x_i \mid y^{k-1})
\end{aligned}
$$

The second expression above follows from the fact that given the state x_j, the state at x_i is independent of all future observations. Constructing the vector $\mathbf{p}(\tau \mid y^n)$ from the above expression yields

$$
\begin{aligned}
\Pr\left(x(\tau) = x_i \mid y^n\right) &= \mathbf{p}_B^T(t_k) \left[\mathrm{diag}(\mathbf{p}_F(t_k^-)])\right]^{-1} \exp[\mathbf{Q}(t_k - \tau)] \mathbf{e}_i \Pr(x(\tau) = x_i \mid y^{k-1}) \\
\mathbf{p}_B^T(\tau) &= \mathbf{p}_B^T(t_k) \left[\mathrm{diag}(\mathbf{p}_F(t_k^-))\right]^{-1} \exp[\mathbf{Q}(t_k - \tau)] \mathrm{diag}(\mathbf{p}_F(\tau)),
\end{aligned}
$$

where $\mathbf{e}_i$ is a vector with 1 as the ith element and 0 for all other elements. The quantity $\mathbf{p}_B^T(t_k) \left[\mathrm{diag}(\mathbf{p}_F(t_k^-)])\right]^{-1}$ corresponds to the element-wise division of the vectors $\mathbf{p}_B^T(t_k)$ and $\mathbf{p}_F(t_k^-)$.

To complete a run of the backward algorithm, it is necessary to store the probability distribution vectors $\mathbf{p}_F(t_k^-)$ generated by the forward algorithm immediately before each observation is made. Storing these values will allow the backward algorithm to calculate $\mathbf{p}_B(t_k)$ for each observation time t_k. To calculate the probability distribution vectors between observation times it is necessary to store additional results from the forward algorithm.

Computation of the backward algorithm is straightforward if the state space is small enough that $\exp[\mathbf{Q}(t_k - \tau)]$ can be directly calculated. If the state space is too large for direction computation of the matrix exponential, more efficient calculation can be made by updating the quantity
$\mathbf{p}_B^T(t_k) \left[\mathrm{diag}(\mathbf{p}_F(t_k^-))\right]^{-1} \exp[\mathbf{Q}(t_k - \tau)]$ via numerical simulation; this quantity is a row vector the can be stored at much lower cost.

1.4 Derivation of Colony Algorithm

The assumptions behind the derivation of the colony algorithm in the main text are: i) each individual molecule is either duplicated at cell division or has an equal probability of being passed to either of the daughter cells; and ii) the division of the cell occurs immediately after the last frame before its daughters are observed. Neither of these assumptions are essential. The first assumption can be relaxed to the requirement that there exists any invertible mapping from the probability distribution function before cell division to the probability distribution function after cell division; the mapping must be invertible for both the forward and backward observers to work. The second assumption is made only for simplicity; the uncertainty of the exact time of division can be modeled by modifying the transition rate matrix $\mathbf{Q}$ in the interframe interval in which it divides to quantify our uncertainty as to whether or not the cell has divided.

Consider a lineage consisting of an ancestral cell A that divides into two sublineages D_1 and D_2, and the division is first observed at time t_d. We wish to express the probability $\Pr\left(x(t_d) = x_i \mid y^{A,D_1,D_2}\right)$ in terms of quantities that can be calculated using the forward and backward algorithms. The derivation proceeds as follows:

$$\begin{aligned}
\Pr\left(x(t_d) = x_i \mid y^{A,D_1,D_2}\right) &= \frac{\Pr(x(t_d) = x_i, y^{D_2} \mid y^{A,D_1})}{\Pr(y^{D_2} \mid y^{A,D_1})} \\
&= \frac{\Pr(y^{D_2} \mid x(t_d) = x_i, y^{A,D_1})\Pr(x(t_d) = x_i \mid y^{A,D_1})}{\Pr(y^{D_2} \mid y^{A,D_1})} \\
&= \Pr(y^{D_2} \mid x(t_d) = x_i)\frac{\Pr(x(t_d) = x_i \mid y^{A,D_1})}{\Pr(y^{D_2} \mid y^{A,D_1})}
\end{aligned}$$

The quantity $\Pr(y^{D_2} \mid x(t_d) = x_i)$ can be expressed using Bayes's Rule as

$$\Pr(y^{D_2} \mid x(t_d) = x_i) = \frac{\Pr(x(t_d) = x_i) \mid y^{D_2})\Pr(y^{D_2})}{\Pr(x(t_d) = x_i)}.$$

The vector form of this expression is

$$\mathbf{p}_{A,D_1,D_2}(t_d) = \frac{\Pr(y^{D_2})}{\Pr(y^{D_2} \mid y^{A,D_1})}\left[\mathrm{diag}\left(\mathbf{p}_{\emptyset}(t_d)\right)\right]^{-1}\mathrm{diag}\left(\mathbf{p}_{D_2}(t_d)\right)\mathbf{p}_{A,D_1}(t_d).$$

The above equation consists of four factors: a constant $\frac{\Pr(y^{D_2})}{\Pr(y^{D_2} \mid y^{A,D_1})}$, a term $\left[\mathrm{diag}\left(\mathbf{p}_{\emptyset}(t_d)\right)\right]^{-1}$ that can be calculated or estimated using the chemical master equation and the invertible cell division map, and two

terms diag $(\mathbf{p}_{D_2}(t_d))$ and $\mathbf{p}_{A,D_1}(t_d)$ that are calculated by running the forward and backward algorithms on the cells when the lineage is divided as in Figure 2.

To calculate this posterior probability along the ancestral cell for $\tau < t_d$, a derivation similar to that of the backward algorithm above yields

$$\mathbf{p}^T_{A,D_1,D_2}(\tau) = \mathbf{p}^T_{A,D_1,D_2}(t_d)\left[\text{diag}(\mathbf{p}_F(t_d^-))\right]^{-1}\exp[\mathbf{Q}(t_d-\tau)]\text{diag}(\mathbf{p}_F(\tau)),$$

so the probability distribution vectors along this interval can be found using the backward algorithm.

Next, we calculate the probability distribution vector $\mathbf{p}^T_{A,D_1,D_2}(\tau)$ for each cell for times greater than t_d, but before the first observation after t_d. We wish to express this quantity in terms of the known quantity $\mathbf{p}^T_{A,D_1,D_2}(t_d)$ and the probabilities calculated in the forward and backward sweep of the colony (cf. Fig. 2). For one branch of the lineage, these quantities are $\mathbf{p}^T_A(\tau)$ and $\mathbf{p}^T_{A,D_1}(\tau)$; for the other branch, they are $\mathbf{p}^T_\emptyset(\tau)$ and $\mathbf{p}^T_{D_2}(\tau)$. Consider the first cell on which the observations y^{A,D_1} were made. By conditioning on the value of the state at t_d, we have the following derivation:

$$\begin{aligned}
\Pr(x_1(\tau)=x_i, \mid y^{A,D_1,D_2}) &= \sum_{x_j\in\mathcal{X}} \Pr(x_1(\tau)=x_i \mid x(t_k)=x_j, y^{A,D_1,D_2})\Pr(x(t_k)=x_j \mid y^{A,D_1,D_2}) \\
&= \sum_{x_j\in\mathcal{X}} \Pr(x_1(\tau)=x_i \mid x(t_k)=x_j, y^{A,D_1})\Pr(x(t_k)=x_j \mid y^{A,D_1,D_2}) \\
&= \sum_{x_j\in\mathcal{X}} \frac{\Pr(x(t_k)=x_j \mid y^{A,D_1,D_2})}{\Pr(x(t_k)=x_j \mid y^{A,D_1})}\Pr(x(t_k)=x_j \mid x(\tau)=x_i, y^A) \\
&\qquad\qquad \times \Pr(x(\tau)=x_i \mid y^{A,D_1}) \\
&= \sum_{x_j\in\mathcal{X}} \frac{\Pr(x(t_k)=x_j \mid y^{A,D_1,D_2})}{\Pr(x(t_k)=x_j \mid y^{A,D_1})}\frac{\Pr(x(\tau)=x_i \mid y^{A,D_1})}{\Pr(x(\tau)=x_i \mid y^{A})} \\
&\qquad\qquad \times \Pr(x(\tau)=x_i \mid x(t_k)=x_j)\Pr(x(t_k)=x_j \mid y^A)
\end{aligned}$$

The vector form of this expression is

$$\mathbf{p}^T_{A,D_1,D_2}(\tau) = \mathbf{p}^T_{A,D_1,D_2}(t_k)\text{diag}\left(\mathbf{p}_A(t_k)\right)\text{diag}\left(\mathbf{p}_{A,D_1}(t_k)\right)^{-1}\exp[\mathbf{Q}(\tau-t_k)]\text{diag}\left(\mathbf{p}_A(\tau)\right)^{-1}\text{diag}\left(\mathbf{p}_{A,D_1}(\tau)\right)$$

For the other cell in the lineage, a similar derivation yields the same result with $\mathbf{p}_\emptyset(.)$ and $\mathbf{p}_{D_2}(.)$ in the place of $\mathbf{p}^T_A(.)$ and $\mathbf{p}^T_{A,D_1}(.)$, respectively. Each of the factors in these expressions can be calculated using

the forward and backward sweep of the colony.

2 Proof of Distinguishability Condition

For simplicity of presentation, we consider only stochastic chemical reaction networks that generate φ-irreducible continuous-time Markov processes [3]. This condition implies that there are no non-reversible transitions in the network. To generalize this result to the case of networks with non-reversible transitions, see [1].

To prove the distinguishability condition expressed in main text Equations 4 and 5, we must first convert the continuous-time Markov chains generated by the reaction network models into discrete-time chains. To do this, we construct, for any $h > 0$, the h-skeleton chain by dividing the time axis into the intevals $[0, h)$, $[h, 2h)$, $\ldots$, and defining the discrete time generator for the model $\mathcal{M}_i$ as $\mathbf{P}_{h,i}$ as $\mathbf{P}_{h,i} = e^{\mathbf{Q_i}h}$. For simplicity we assume $i = 1$ or $i = 2$; the proof can be extended to any finite number of models. For the case of perfect observation, each state $\mathbf{x}$ generates a single output y. We will denote by $\mathcal{Y}_j$ the set of all states that generate the output y_j. We will denote the output at stage n as y_{j_n}, and define $y^n = \{y_{j_1}, y_{j_2} \ldots y_{j_n}\}$ as the sequence of outputs up to stage n. The recurrence properties of the CTMC are preserved by the h-skeleton chain for any $h > 0$ [3].

We must also convert the distinguishability condition to discrete time. The discrete-time equivalent to Eq. 1 main text is: for all $\epsilon > 0$ and $\alpha < 1$, there exists a positive N such that for all $n > N$,

$$\Pr(\omega : D(\omega) = 0 \mid \|\omega\| = n) < \epsilon,$$

where $D(\omega) = 1$ if $\Pr(\mathcal{M}_1 \mid \mathbf{h}(\omega)) > \alpha$ and 0 otherwise.

For any discrete-time h-skeleton chain, we define the extended h-skeleton chain by extending the state space to include both the current and previous state. The state space of the extended h-skeleton chain is therefore $\mathcal{X} \times \mathcal{X}$ and we denote the state of this chain at stage n by (x_n, x_{n-1}).

Each extended h-skeleton chain has a unique steady-state distribution $\mathbf{p}_{ss,h}$. At the steady-state, we can consider the probability $\Pr_{ss,h}(\rho_{j(n),j(n+1)} \mid y_{j(n)}, y_{j(n-1)})$, which is the probability of a transition $\rho_{j(n),j(n+1)}$ from the output y_{j_n} to $y_{j_{n+1}}$, given that the output at the previous stage was $y_{j_{n-1}}$. This

quantity can be calculated from $\mathbf{p}_{ss,h}$ as

$$\Pr_{ss,h}(\rho_{j(n),j(n+1)} \mid y_{j(n)}, y_{j(n-1)}) = \frac{\sum_{x_n \in \mathcal{Y}_n} \sum_{x_{n-1} \in \mathcal{Y}_{n-1}} \Pr_{ss,h}(\rho_{j(n),j(n+1)} \mid x_n, x_{n-1}) \Pr_{ss,h}(x_n, x_{n-1})}{\sum_{x_n \in \mathcal{Y}_n} \sum_{x_{n-1} \in \mathcal{Y}_{n-1}} \Pr_{ss,h}(x_n, x_{n-1})}.$$

We will say that two h-skeleton chains $\mathbf{P}_{h,1}$ and $\mathbf{P}_{h,2}$ are *probabilistically output indistinguishable* (POI) if their extended chains have the property

$$\Pr_{ss,h,1}(\rho_{j(n),j(n+1)} \mid y_{j(n)}, y_{j(n-1)}) = \Pr_{ss,h,2}(\rho_{j(n),j(n+1)} \mid y_{j(n)}, y_{j(n-1)})$$

for all choices of $\rho_{j(n),j(n+1)}$, $y_{j(n)}$, and $y_{j(n-1)}$.

Lemma: Two extended h-skeleton chains $\mathbf{P}_{h,1}$ and $\mathbf{P}_{h,2}$ are distinguishable if and only if they are POI.

Without loss of generality, assume that $\mathcal{M}_1$ is the true model. Let $\epsilon_1, \epsilon_2 > 0$. By the ergodic theorem, there exists an $m_1 > 0$ such that

$$\Pr\left[y^{m_1} : \| \Pr(\rho_{j(m),j(m+1)} \mid y^{m_1}) - \Pr_{ss,h,1}(\rho_{j(m),j(m+1)} \mid y_{j(m)}, y_{j(m-1)}) \| > \frac{\epsilon_1}{2}\right] < \epsilon_2$$

(Necessity) Suppose that $\mathbf{P}_{h,1}$ and $\mathbf{P}_{h,2}$ are not POI. Let y^{m_2} be a sequence of m_2 observations that follows the original m_1 observations. Consider the first$y_{j(m_1+1)}$, the first of the sequence of m_2 observations. Because the two models are POI, it follows that the likelihood ratio of $\mathcal{M}_1$ with respect to $\mathcal{M}_2$ must be bounded by

$$\frac{p_1 - \epsilon_2}{p_1} < \frac{\Pr(y_{j(m_1+1)} \mid \mathcal{M}_1, y_{j(m_1)}, y_{j(m_1-1)})}{\Pr(y_{j(m_1+1)} \mid \mathcal{M}_2, y_{j(m_1)}, y_{j(m_1-1)})} < \frac{p_1}{p_1 - \epsilon_2}, \tag{10}$$

where $p_1 = \Pr_{ss,h,1}(\rho_{j(m),j(m+1)} \mid y_{j(m)}, y_{j(m-1)})$.

Let $p_i = \Pr_{ss,h,1}(\rho_{j(m+i-1),j(m+i)} \mid y_{j(m+i-1)}, y_{j(m+1-2)})$.Because the h-skeleton chain converges geometrically to steady-state at a rate defined by the second-largest eigenvalue modulus γ [4, Ch. 6], extending the above equation from $y_{j(m_1+1)}$ to y^{m_2} yields

$$\prod_{i=1}^{m_2} \frac{p_i - \gamma^{i-1}\epsilon_2}{p_i} < \frac{\Pr(y^{m_2}) \mid \mathcal{M}_1, y_{j(m_1)}, y_{j(m_1-1)})}{\Pr(y^{m_2}) \mid \mathcal{M}_2, y_{j(m_1)}, y_{j(m_1-1)})} < \prod_{i=1}^{m_2} \frac{p_i}{p_i - \gamma^{i-1}\epsilon_2}. \tag{11}$$

Because m_2 is finite, there must be a non-zero minimum among the p_i; denote this minimum by p_*.

Replacing each p_i with p_* loosens the bounds on the likelihood ratio. Furthermore, we can replace the finite geometric series of length m_2 with its infinite sum while preserving the inequalities, and discard all terms of order $\mathcal{O}(\epsilon_2^2)$. These operations yield

$$\frac{p_*^{m_2} - \frac{\epsilon_2}{1-\gamma}}{p_*^{m_2}} < \frac{\Pr(y^{m_2}) \mid \mathcal{M}_1, y_{j(m_1)}, y_{j(m_1-1)})}{\Pr(y^{m_2}) \mid \mathcal{M}_2, y_{j(m_1)}, y_{j(m_1-1)})} < \frac{p_*^{m_2}}{p_*^{m_2} - \frac{\epsilon_2}{1-\gamma}}. \tag{12}$$

Denote the likelihood ratio of $\mathcal{M}_1$ to $\mathcal{M}_2$ at stage m_1 as β. For any m_2, there exists ϵ_2 sufficiently small so that $\xi = \frac{p_*^{m_2} - \frac{\epsilon_2}{1-\gamma}}{p_*^{m_2}} > 0$. Thus, after $m_1 + m_2$ stages, the likelihood ratio must be between $\xi\beta$ and β/ξ, and these two quantities are bounded strictly between zero and one even as m_2 becomes arbitrarily large. Therefore, the probability of $\mathcal{M}_1$ can never be greater than $(1 + \xi\beta)^{-1}$. Thus, by choosing $\alpha > (1 + \xi\beta)^{-1}$, the discrete-time distinguishability condition cannot be satisfied, and thus the two models are indistinguishable.

(Sufficiency) Suppose that $\mathbf{P}_{h,1}$ and $\mathbf{P}_{h,2}$ are POI. Then there exists a triple $\rho_{j(n),j(n+1)}, y_{j(n)}, y_{j(n-1)}$ such that $\Pr_{ss,h,1}(\rho_{j(n),j(n+1)} \mid y_{j(n)}, y_{j(n-1)}) \neq \Pr_{ss,h,2}(\rho_{j(n),j(n+1)} \mid y_{j(n)}, y_{j(n-1)})$.

Consider the likelihood function:

$$f(\mathbf{a}) = \prod_{(y,y')} \left[\begin{pmatrix} n_{y,y'} \\ k_{\rho_1,y,y'}, \ldots, k_{\rho_\ell,y,y'} \end{pmatrix} \prod_{i=1}^{\ell} \left(\frac{k_{\rho_i,y,y'}}{n_{y,y'}} + a_{i,y,y'} \right)^{k_{\rho_i,y,y'}} \right], \tag{13}$$

where $n_{y,y'}$ denotes the number of transitions from output y to output y', $k_{\rho_i,y,y'}$ denotes the number of times that this transition is followed by the output transition ρ_i, and $a_{i,y,y'}$ denotes the observed deviation from the expected fraction of occurrences of $k_{\rho_i,y,y'}$.

Denote the multinomial coefficient associated with (y, y') by $M_{y,y'}$. Taking the logarithm of the preceding function yields

$$\log f(\mathbf{a}) = \log \sum_{(y,y')} \left(M_{y,y'} + n_{y,y'} \sum_{i=1}^{m} \frac{k_{e_i q e' q'}}{n_{y,y'}} \log \left(\frac{k_{\rho_i,y,y'}}{n_{y,y'}} + a_{\rho_i,y,y'} \right) \right). \tag{14}$$

Consider the function

$$g(\mathbf{a}) = \log \sum_{(y,y')} \left(n_{y,y'} \sum_{i=1}^{m} \frac{k_{e_i q e' q'}}{n_{y,y'}} \log \left(\frac{k_{\rho_i,y,y'}}{n_{y,y'}} + a_{\rho_i,y,y'} \right) \right). \tag{15}$$

Take the gradient and Hessian of $g(\mathbf{a})$. Based on these values, we determine that the function has a global maximum at $\mathbf{a} = 0$ and that, because the Hessian is everywhere positive, the function is concave. Also, we note that the function is locally Lipschitz continuous about $\mathbf{a} = 0$. Therefore, there exists a constant L_1 such that if all $a_{\rho_i,y,y'} < \epsilon^*/L_1$, such that the value of $g(\mathbf{0}) - g(\mathbf{a})$ is no greater than ϵ^*.

Since, by assumption, $\mathcal{M}_1$ is the true model, by the ergodic theorem, for all $\epsilon > 0$ there exists $N \in \mathbb{N}$ such that $n > N$ implies

$$\Pr(t : \exists k_{\rho_i,y,y'} \text{ such that } \left\| \frac{k_{\rho_i,y,y'}}{n_{y,y'}} - \Pr_{ss,h,1}(\rho_i \mid y, y') \right\| > \frac{\epsilon^*}{L_1}) < \epsilon. \tag{16}$$

Therefore the local Lipschitz continuity demonstrates that $g(\mathbf{0}) - g(\mathbf{a}) < \epsilon$ for $\mathcal{M}_1$.

Let $\mathbf{x}$ be a point when at least one co-ordinate $a_{\rho_i,y,y'}$ in $\mathbf{x}$ is bounded away from zero, i.e., let $a_{\rho_i,y,y'} > K$. Because ∇g is always positive away from zero, the difference $g(\mathbf{0}) - g(\mathbf{x})$ must also be bounded away from zero, as it is equal to the quantity $\int_{\mathbf{x}}^{\mathbf{0}} \nabla g(\phi) d\phi$, which is the path integral of a function that is always positive over a length of at least K. Therefore the difference $g(\mathbf{0}) - g(\mathbf{x}) \geq K > 0$.

Because the extended h-skeleton chains $\mathbf{P}_{h,1}$ and $\mathbf{P}_{h,2}$ are POI, $\mathbf{P}_{h,2}$ contains at least one $a_{iqe'q'}$ in $\mathbf{x}$ bounded away from zero. It follows that $g(\mathbf{0}) - g(\mathbf{x}) \geq K^*$. By choosing $\epsilon < K^*$, we can ensure that $g(\mathbf{a}) - g(\mathbf{x})$ is positive. The log of the likelihood ratio of $\mathbf{P}_{h,1}$ with respect to $\mathbf{P}_{h,2}$ can be bounded by

$$L(\mathbf{P}_{h,1}) > \frac{g(\mathbf{a})}{g(\mathbf{x})} > \min\left(n_{y,y'}\right)\left(g(\mathbf{a}) - g(\mathbf{x})\right). \tag{17}$$

Because the h-skeleton chain is positive recurrent, the ratio of $\min\left(n_{y,y'}\right)$ to n, the total number of stages, is positive, it follows that $L(\mathbf{P}_{h,1}) \to \infty$ as $n \to \infty$. Choose n^* such that $L > \frac{\alpha(1+\epsilon)}{1-\alpha(1+\epsilon)}$. Then for all ϵ and α, there exists an n such that $D_\alpha(st) = 1$. Therefore if the extended h-skeleton chains $\mathbf{P}_{h,1}$ and $\mathbf{P}_{h,2}$ are POI, then they are distinguishable.

We now need to convert the discrete-time result back to continuous time. If the extended h-skeleton chains $\mathbf{P}_{h,1}$ and $\mathbf{P}_{h,2}$ are POI for one choice of h, they are POI for any choice of h. Choose a very small h and consider the quantity $\Pr_{ss,h,1}(\rho_{j(n),j(n+1)} \mid y_{j(n)}, y_{j(n-1)}) - \Pr_{ss,h,1}(\rho_{j(n-1),j(n)} \mid y_{j(n-1)}, y_{j(n-2)})$.

If $y_{j(n)} \neq y_{j(n-1)}$, then the dwell time of the output $y_{j(n)}$ can be made arbitrarily close to zero and thus, if the h-skeleton chains are POI, we require that $r_{ss,1}(y, 0) = r_{ss,2}(y, 0)$ for all outputs y. If $y_{j(n)} = y_{j(n-1)}$, then the dwell time is some value $\tau > 0$ and, if the h-skeleton chains are POI, we require that $r_{ss,1}(y, \tau) = r_{ss,2}(y, \tau)$ for all outputs y and all dwell times τ.

In the more realistic case of periodic observations at a fixed interval h (where h is the frame rate, in the range of 10-100 minutes), the condition for distinguishability is equivalent because the property of probabilistic output distinguishability holds for all $h > 0$. In the case of periodic observations, the proof only requires that the POI property holds for one h (the frame rate) and thus it follows directly from the proof for continual observation. In this case, to determine the practical distinguishability of models, it is useful to also calculate quantities like the Fisher Information Matrix [5] to determine how quickly the pair of models can be distinguished using a given frame rate. The questions of determining an optimal frame rate or optimal non-periodic observation strategy are outside the scope of this paper.

References

1. Thorsley D, Teneketzis D (2005) Diagnosability of stochastic discrete-event systems. IEEE Transactions on Automatic Control 50: 476-492.

2. Munsky B, Khammash M (2006) The finite state projection algorithm for the solution of the chemical master equation. The Journal of Chemical Physics 124: 044104.

3. Tuominen P, Tweedie RL (1979) Exponential decay and ergodicity of general markov processes and their discrete skeletons. Advances in Applied Probability 11: 784-803.

4. Brémaud P (1999) Markov Chains: Gibbs Fields, Monte Carlo Simulation and Queues. New York: Springer.

5. Komorowski M, Costa MJ, Rand DA, Stumpf MPH (2011) Sensitivity, robustness, and identifiability in stochastic chemical kinetics models. Proceedings of the National Academy of Sciences 108: 8645-8650.
